# Salivary miR-21 and miR-31 in Oral Squamous Cell Carcinoma: Molecular Pathogenesis, Diagnostic Significance, and Translation to Isothermal RPA

**DOI:** 10.3390/cells15191799

**Published:** 2026-10-01

**Authors:** Jyotsna S. Ranbhise, Manish Kumar Singh, Hyeong Rok Yun, Sunhee Han, Sung Soo Kim, Insug Kang

**Affiliations:** 1 Department of Biochemistry and Molecular Biology, School of Medicine, Kyung Hee University, Seoul 02447, Republic of Korea; jogm25@khu.ac.kr (J.S.R.); manishbiochem@gmail.com (M.K.S.); foryou018@naver.com (H.R.Y.); sunheehan@khu.ac.kr (S.H.); 2 Biomedical Science Institute, Kyung Hee University, Seoul 02447, Republic of Korea; 3 Department of Biomedical Science, Graduate School, Kyung Hee University, Seoul 02447, Republic of Korea

**Keywords:** oral squamous cell carcinoma, salivary diagnostics, miR-21, miR-31, recombinase polymerase amplification, isothermal amplification, point-of-care testing, microRNA, early detection, risk stratification

## Abstract

**Highlights:**

**What are the main findings?**

- Salivary miR-21 and miR-31 are biologically relevant candidate biomarkers for OSCC, reflecting partly distinct processes related to oncogenic survival, epithelial dysregulation, and invasive behavior.
- Their diagnostic translation is limited by substantial methodological heterogeneity in saliva collection, RNA recovery, normalization, and diagnostic threshold determination.

**What are the implications of the main findings?**

- Adapting mature miRNA detection to Recombinase Polymerase Amplification (RPA) requires overcoming the mismatch between short miRNA targets and conventional RPA primer architecture through suitable target-extension strategies.
- Clinical translation will require standardized, contamination-controlled sample-to-answer systems validated in early-stage OSCC and clinically relevant comparator populations, with salivary miRNA testing positioned as a risk-stratification or triage adjunct rather than a replacement for biopsy.

**Abstract:**

Oral Squamous Cell Carcinoma (OSCC) is often diagnosed at advanced stages despite the oral cavity’s anatomical accessibility, highlighting the need for molecular adjuncts that can identify disease-associated changes before overt morphological progression. This review evaluates the biological roles and salivary diagnostic potential of microRNA-21 (miR-21) and miR-31, two miRNAs repeatedly associated with OSCC pathogenesis and progression. We examine current clinical evidence supporting their use as salivary biomarkers and discuss major sources of methodological variability, including differences in saliva collection, RNA recovery, normalization, and diagnostic thresholds. We pay particular attention to the transition from laboratory-based RT-qPCR to Recombinase Polymerase Amplification (RPA) for decentralized detection. Although RPA offers rapid, low-temperature amplification with reduced hardware requirements, mature miRNAs present a key technical challenge because their short length requires prior target extension or conversion. Successful chairside translation will depend on standardized sample processing, robust target-specific amplification, contamination control, and validation in clinically relevant early-stage and comparator populations.

## 1. Introduction

Oral Squamous Cell Carcinoma (OSCC) represents a significant global health burden, accounting for approximately 90% of all oral malignancies [1]. Despite the oral cavity’s anatomical accessibility, which theoretically allows for direct visual inspection and tactile assessment, OSCC is frequently diagnosed at advanced clinical stage [2,3,4]. The clinical implications of this diagnostic delay are profound: localized Stage I and II tumors are associated with five-year survival rates exceeding 90%, whereas detection at Stage III or IV corresponds with a substantial reduction in survival to less than 30% [5,6,7,8]. Although precise figures vary by region and cohort, many patients present with advanced disease, suggesting that the established screening model, which relies primarily on conventional oral examination, has limited sensitivity for detecting molecular changes before overt morphological disease becomes apparent. Histopathological biopsy remains the definitive diagnostic standard, yet it is inherently invasive and typically reactive, performed only after a suspicious lesion has become clinically obvious [9,10]. Consequently, objective molecular tools could complement conventional examination by identifying molecular alterations before advanced disease becomes clinically apparent.

In this context, saliva has emerged as a promising medium for local liquid biopsy due to its direct anatomical proximity to the oral tumor microenvironment [11]. As a biofluid in constant contact with the malignant epithelium, saliva serves as a noninvasive reservoir for tumor-associated molecular signals, including nucleic acids, proteins, extracellular vesicles, and metabolites [12,13,14]. Unlike blood-based biopsies, salivary collection is repeatable, low-cost, and well-suited to standard dental clinical settings [14,15,16]. Furthermore, saliva contains both locally derived and systemically influenced molecular signals, offering a unique diagnostic window. However, while saliva is biologically rich, its composition is influenced by diverse local factors and should not be regarded as a perfectly tumor-specific medium without rigorous analytical validation.

Among the molecular signatures found in saliva, microRNAs (miRNAs) are of particular diagnostic interest [17]. These small, non-coding RNA molecules (typically 19–24 nucleotides) function as post-transcriptional regulators of gene expression [18,19]. Their profiles are often significantly altered during tumorigenesis, reflecting the loss of cellular homeostatic control. A key advantage of miRNAs over other RNA species is their relative biophysical stability; they are frequently sequestered within lipid-bilayer exosomes or complexed with proteins, which protects them from enzymatic degradation [20,21,22]. This stability enables reliable measurement of miRNA concentrations across diverse media, including tissue, plasma, and saliva, making them attractive candidates for biomarker development [23,24].

The selection of miR-21 and miR-31 as the primary subjects of this review is substantiated by consistent systematic evidence identifying them as the most reproducible and accurate salivary indicators of OSCC [25,26]. Meta-analyses of the salivary transcriptomic landscape have repeatedly ranked miR-21 and miR-31 among the biomarkers with the highest diagnostic performance, frequently reporting Area Under the Curve (AUC) values exceeding 0.85 and sensitivities above 80% [27,28,29]. Mechanistically, these two markers provide a complementary molecular profile of the disease: miR-21 is a primary driver of oncogenic survival and proliferative signaling via the PTEN/PI3K/Akt axis. At the same time, miR-31 is a critical player of epithelial dysregulation and invasive behavior [30,31,32]. By focusing on this dual-marker approach, this review evaluates a molecular framework that captures both the proliferative and invasive dimensions of the malignant phenotype.

However, a significant translational gap remains between biomarker discovery and clinical deployment. Although RT-qPCR routinely achieves high-sensitivity detection, this widely used reference method often requires multi-step RNA purification, reverse transcription, and precise thermal cycling, making the workflow incompatible with immediate chairside risk stratification [33,34,35]. One potential technological solution is the implementation of isothermal amplification strategies, specifically Recombinase Polymerase Amplification (RPA). RPA represents a potential alternative to PCR-based hardware because it operates at a constant, low temperature (37–42 °C) [36]. This technology is theoretically compatible with portable readouts, which could move molecular sensing from the laboratory to the clinical point of care. However, a significant scientific hurdle remains: mature miRNAs are unusually difficult RPA targets because their length (~22 nt) is shorter than the typical primers required for stable recombinase-mediated homology search [37,38].

This review critically evaluates the clinical and technical framework required to bridge this gap. We assess the molecular roles of miR-21 and miR-31 and the current clinical evidence supporting their value as adjunctive diagnostic markers in saliva. Furthermore, we analyze the causes of methodological variability between studies and the feasibility of adapting short-miRNA detection to isothermal RPA. Ultimately, we outline the requirements for a standardized, chairside risk-stratification platform designed to triage patients with existing suspicious oral lesions.

### Review Strategy and Literature Selection

To improve the transparency and reproducibility of this review, a structured literature search was conducted between 12 July and 20 August 2026 using PubMed/MEDLINE, Scopus, and the Web of Science Core Collection.

The search strategy was designed around three major themes: (i) salivary miR-21 and miR-31 in Oral Squamous Cell Carcinoma (OSCC), (ii) molecular mechanisms associated with miR-21 and miR-31 in oral carcinogenesis, and (iii) isothermal amplification and Recombinase Polymerase Amplification (RPA) for short-RNA detection. Combinations of Medical Subject Headings (MeSH) terms and free-text keywords were used, including “salivary microRNA,” “miR-21,” “miR-31,” “oral squamous cell carcinoma,” “OSCC,” “oral cancer,” “diagnostic accuracy,” “sensitivity,” “specificity,” “ROC,” “AUC,” “isothermal amplification,” “Recombinase Polymerase Amplification,” and “RPA.”

Studies were considered eligible if they: (a) evaluated salivary miR-21 and/or miR-31 in human OSCC cohorts; (b) reported diagnostic or clinically relevant biomarker data; (c) investigated the molecular roles of miR-21 or miR-31 in OSCC or closely related oral epithelial models; or (d) examined technical approaches relevant to miRNA conversion, isothermal amplification, or RPA-based detection. Peer-reviewed systematic reviews and meta-analyses were also consulted for contextual synthesis and reference tracing.

Studies were excluded if they were case reports, editorials, or conference abstracts without sufficient primary data; focused exclusively on non-oral malignancies without relevance to OSCC; or evaluated blood, urine, or other non-salivary biofluids without a salivary diagnostic component. Tissue-based and cell-based studies were retained only when directly relevant to the mechanistic evaluation of miR-21 or miR-31 in OSCC.

Records were screened in two stages, first by title and abstract and subsequently by full-text assessment. For the clinical biomarker synthesis, data were extracted on sample size, OSCC stage, comparator group, saliva type, collection protocol, RNA extraction method, detection platform, normalization strategy, expression result, diagnostic threshold, sensitivity, specificity, AUC, and independent validation. These variables were used to construct Table 1 and to evaluate methodological heterogeneity across the included clinical studies.

## 2. miR-21 in OSCC: Molecular Pathogenesis

MicroRNA-21 (miR-21) was among the first mammalian miRNAs identified and is located on chromosome 17q23.2 within the 11th intron of the *VMP1* (vacuole membrane protein 1) gene [45,46]. miR-21 is widely classified as an oncogenic microRNA because its overexpression has been associated with suppression of multiple tumor-suppressive pathways across malignancies. In OSCC and oral epithelial models, elevated miR-21 expression has been repeatedly reported and has been linked to proliferative, survival, and invasive phenotypes [47,48]. Specifically, in high-risk populations, exposure to areca-derived alkaloids, particularly arecoline, has been associated with the transcriptional upregulation of the *VMP1* promoter, which may promote the accumulation of primary miR-21 (pri-miR-21) transcripts [49,50]. These transcripts undergo canonical miRNA processing through Drosha/DGCR8-mediated cleavage, nuclear export, and subsequent Dicer-dependent maturation. This canonical processing pathway generates mature miR-21, which is frequently elevated in specimens of OSCC [51,52,53,54,55].

### 2.1. Targeted Suppression of Tumor Suppressor Networks

miR-21 exerts its oncogenic influence primarily by post-transcriptionally silencing genes that normally inhibit malignant transformation. PTEN (Phosphatase and Tensin Homolog) is one of the most extensively characterized miR-21 targets in cancer biology. Direct miR-21–PTEN interactions have been functionally demonstrated in several tumor models, whereas OSCC studies additionally support an association between elevated miR-21, reduced PTEN activity, and dysregulated PI3K/Akt signaling [56,57,58]. Thus, the PTEN/PI3K/Akt axis represents a biologically plausible and clinically relevant pathway in OSCC, although the strength of direct miR-21 target validation differs among individual oral cancer studies.

Beyond the PTEN axis, miR-21 targets Programmed Cell Death 4 (PDCD4), which under physiological conditions acts as a translational inhibitor of the eukaryotic initiation factor 4A (eIF4A). Functional regulation of the miR-21/PDCD4 axis has been demonstrated in several epithelial and head-and-neck malignancies, while oral cancer studies support its relevance to proliferation and survival [59,60,61,62]. In OSCC specifically, stronger functional evidence exists for regulation of RECK (Reversion-inducing Cysteine-rich Protein with Kazal motifs), where manipulation of miR-21 has been associated with changes in RECK expression and invasive behavior [32,63,64]. TPM1 has also been implicated in miR-21-associated tumor biology, although some mechanistic evidence for this interaction is derived from broader cancer models rather than direct OSCC-specific functional studies [65,66].

Collectively, these findings support the involvement of miR-21 in tumor-suppressor network disruption in OSCC, while also emphasizing that not all reported miR-21 targets have been validated to the same degree within oral squamous carcinoma itself.

### 2.2. Signaling Hyperactivation: The PI3K/Akt/mTOR Pathological Axis

miR-21-mediated suppression of PTEN and PDCD4 can sustain activation of several interconnected signaling cascades, most notably the PI3K/Akt/mTOR axis. Without sufficient PTEN-mediated phosphatase activity, PIP3 accumulates at the plasma membrane, facilitating downstream Akt activation. This signaling axis regulates multiple downstream processes involved in cell-cycle progression and apoptosis evasion [67,68]. For example, enhanced Akt signaling can promote MDM2 phosphorylation, facilitating nuclear translocation and proteasomal degradation of the p53 tumor suppressor and potentially neutralizing intrinsic apoptotic triggers [69,70]. Additionally, this axis is associated with Cyclin D1 stabilization through inhibition of GSK3β, which may accelerate the G1/S phase transition and promote mitotic expansion [71,72]. These downstream interactions are well established in cancer biology; however, not every component of this cascade has been directly demonstrated through miR-21 manipulation in OSCC. They should therefore be interpreted as mechanistic pathways potentially linking miR-21-associated PTEN suppression to malignant behavior rather than as uniformly proven miR-21-dependent events in oral cancer.

Evidence in oral malignancy also suggests that this hyperactive axis may influence the Epithelial-to-Mesenchymal Transition (EMT) by modulating transcription factors such as Snail and Slug [73,74]. However, some molecular steps within this proposed miR-21–EMT relationship are supported primarily by broader epithelial cancer models rather than direct OSCC-specific functional experiments.

### 2.3. Functional Remodeling on the OSCC Phenotype

The biochemical effects associated with miR-21 promote cellular survival, proliferation, and invasive behavior. Enhanced cell survival may be maintained through the concomitant downregulation of pro-apoptotic factors such as BAX and the upregulation of anti-apoptotic BCL-2, creating a cellular environment more resistant to DNA-damage-induced death [75]. However, the extent to which these alterations result directly from miR-21 manipulation in OSCC varies among studies.

An important functional consequence associated with miR-21 activity is enhanced invasive and metastatic potential [55]. More direct OSCC evidence supports a role for miR-21 in invasive behavior through regulation of tumor-suppressive targets such as RECK. miR-21-associated signaling has also been linked to altered activity of Matrix Metalloproteinases, particularly MMP-2 and MMP-9, which participate in extracellular-matrix degradation and basement-membrane remodeling [76,77,78,79]. Nevertheless, some mechanistic links between miR-21 and individual MMP pathways derive from non-OSCC experimental systems. These observations therefore support a role for miR-21 in extracellular-matrix remodeling but do not establish a single universal miR-21→MMP pathway across all OSCC models.

Consistent with these experimental observations, elevated miR-21 has been associated clinically with invasive disease and lymph-node involvement. However, associations with metastatic behavior should be distinguished from direct experimental demonstration that miR-21 alone is sufficient to drive metastasis.

### 2.4. Clinical Significance: Staging, Prognosis, and Therapeutic Resistance

Clinically, elevated miR-21 expression has been associated with advanced TNM (Tumor, Node, Metastasis) stage and regional lymph node involvement (N+) in several studies [80,81]. Several clinical studies have linked elevated miR-21 expression to shorter disease-free and overall survival compared with patients with low expression.

Furthermore, miR-21 is increasingly investigated as a potential modulator of treatment resistance. Evidence regarding treatment resistance should be interpreted according to treatment modality. Recent functional work in OSCC provides direct evidence that miR-21-5p can influence radiation-response pathways, supporting a mechanistic contribution to radioresistance. In contrast, the relationship between miR-21 and cisplatin resistance is supported by a broader and more heterogeneous literature encompassing oral, head-and-neck, and other cancer models [82,83,84]. These observations suggest that miR-21 may participate in treatment resistance mechanisms, including survival signaling through pathways such as Akt, but treatment specific effects should not be generalized across modalities without direct validation.

These findings support a potential prognostic role for miR-21, although the current heterogeneity across cohorts and analytical methods, discussed in Section 5, limits immediate clinical application [85].

Overall, the evidence supporting individual molecular targets, metastatic effects, and treatment-response pathways ranges from direct functional validation in OSCC to associations and mechanistic inference from broader cancer models. This distinction should be considered when interpreting miR-21 as a translational biomarker.

## 3. miR-31 in OSCC: Molecular Pathogenesis

MicroRNA-31 (miR-31) is encoded on chromosome 9p21.3, a genomic region frequently associated with diverse malignancies [86,87]. In OSCC, miR-31 is consistently identified as one of the most highly overexpressed non-coding RNAs [42]. While its functional role is context-dependent in other tissues, it appears to be predominantly oncogenic in the oral epithelium, where it has been associated with the modulation of cellular responses to hypoxia, epithelial instability, and tissue infiltration [88]. Functional studies in oral cancer support roles for miR-31 in pathways related to epithelial plasticity, hypoxic adaptation, proliferation, migration, and invasion, although the strength of evidence varies according to the specific molecular interaction examined.

### 3.1. Expression Profiles Across Oral Carcinogenesis

miR-31 expression has been reported to be significantly elevated in OSCC relative to normal oral mucosa and has also been detected at altered levels in several Oral Potentially Malignant Disorders (OPMDs) [25,89]. miR-31 upregulation has also been reported in dysplastic lesions associated with higher malignant potential [90,91,92]. These observations suggest that miR-31 dysregulation may occur relatively early in oral carcinogenesis, although its ability to predict malignant transformation remains uncertain prospectively. Importantly, cross-sectional differences between normal mucosa, OPMDs, and established OSCC do not themselves demonstrate prospective prediction of malignant transformation. Longitudinal evidence remains comparatively limited.

As the disease advances, some studies have reported higher miR-31 expression at the invasive front of the tumor compared to the central tumor mass, although longitudinal evidence for this expression trajectory remains limited [93,94]. These findings are consistent with a possible role in invasive progression but should not be interpreted as evidence of a universal temporal increase in miR-31 throughout disease progression.

### 3.2. Target-Mediated Signaling: HIF-1α, EMT, and Cytoskeletal Remodeling

miR-31 may mechanistically contribute to oral malignancy by repressing genes essential for homeostatic epithelial regulation. One well-characterized target is the Factor Inhibiting HIF-1 (FIH-1) [95]. The mature miR-31 sequence binds to the 3′ untranslated region (UTR) of the FIH-1 mRNA, leading to its translational repression. Under physiological conditions, FIH-1 suppresses the activity of Hypoxia-Inducible Factor 1-alpha (HIF-1α); consequently, the miR-31-mediated suppression of FIH-1 has been associated with enhanced HIF-1α activity, potentially influencing hypoxia-responsive transcription and metabolic adaptation [96,97].

The LATS2/Hippo pathway represents another important mechanistic axis. Direct functional evidence in OSCC models supports regulation of LATS2-related Hippo signaling by miR-31, including effects on cellular proliferation, migration, invasion, and EMT-associated phenotypes [98,99,100]. Related miR-31–LATS2 interactions have also been described in other squamous carcinomas; these studies provide supportive mechanistic context but should be distinguished from OSCC-specific validation.

miR-31 dysregulation has also been associated with changes related to the Epithelial-to-Mesenchymal Transition (EMT) in oral malignancy, including altered expression of epithelial and mesenchymal markers and cytoskeletal remodeling [101,102,103]. Specifically, in oral epithelial models, these shifts are often characterized by the downregulation of E-cadherin and the reciprocal upregulation of N-cadherin and Vimentin. In OSCC models, functional manipulation of the miR-31/LATS2 axis has been associated with these EMT-related marker changes, providing direct support for a role of miR-31 in epithelial remodeling.

Furthermore, activation of the RhoA/ROCK1 signaling pathway has been linked to miR-31-mediated cytoskeletal reorganization, including the formation of lamellipodia and filopodia [104]. However, evidence for this specific miR-31–RhoA/ROCK1 relationship is supported more substantially by broader cancer and cell-migration literature than by direct OSCC functional studies. These observations are consistent with a potential role for miR-31 in enhancing cellular migration and tissue infiltration [105].

### 3.3. Functional and Clinical Significance: Invasion and Metastatic Potential

Mechanistically, miR-31 expression has been associated with increased migration, extracellular matrix remodeling, and the activity of Matrix Metalloproteinases, specifically MMP-2 and MMP-9 [106,107,108]. Functional OSCC studies support a role for miR-31 in migration and invasion. Manipulation of miR-31-related pathways has been associated with changes in invasive capacity and with expression of extracellular matrix remodeling molecules, including MMP-2 and MMP-9. These findings provide stronger mechanistic support for an invasive role of miR-31 in OSCC. Clinically, some studies report associations between elevated miR-31 expression and advanced clinicopathological characteristics, such as increased tumor thickness (T3-T4) and the presence of regional lymph node metastasis (N+) [107]. However, experimental evidence of enhanced invasion should be distinguished from clinical proof that miR-31 independently drives regional or distant metastasis. Such clinical associations are consistent with, but do not establish, a causal role in metastatic progression.

Because elevated miR-31 expression has been reported across dysplastic and invasive lesions, it has been proposed as a candidate molecular indicator of malignant transformation [40,89,109]. However, prognostic evidence remains heterogeneous. While elevated miR-31 levels in tissue and saliva correlate with aggressive disease behavior in specific cohorts, these findings do not yet establish miR-31 as an independent marker of metastatic risk or local recurrence. Current evidence therefore supports the biological relevance of miR-31 and its continued evaluation as a diagnostic and risk-stratification biomarker, but does not establish it as an independent prognostic marker.

### 3.4. Rationale for the Dual Evaluation of miR-21 and miR-31

The rationale for evaluating miR-21 and miR-31 as a combined salivary panel stems from their potential biological complementarity. miR-21 is strongly associated with survival and proliferative signaling through the regulation of the PTEN/PI3K/Akt axis, whereas miR-31 has been associated with epithelial dysregulation and invasive behavior. This biological complementarity provides a rationale for evaluating the two markers within the same diagnostic framework. It does not, however, establish diagnostic synergy or demonstrate that a combined miR-21/miR-31 panel outperforms either marker individually. Evidence of superior combined performance would require direct comparison of miR-21 alone, miR-31 alone, and the combined model within the same clinical cohort, followed by validation in an independent population. Accordingly, the present review considers miR-21 and miR-31 jointly because they represent biologically relevant but partially distinct OSCC-associated miRNAs, rather than because superior diagnostic performance of a dual-marker panel has already been established.

**Figure 1:**
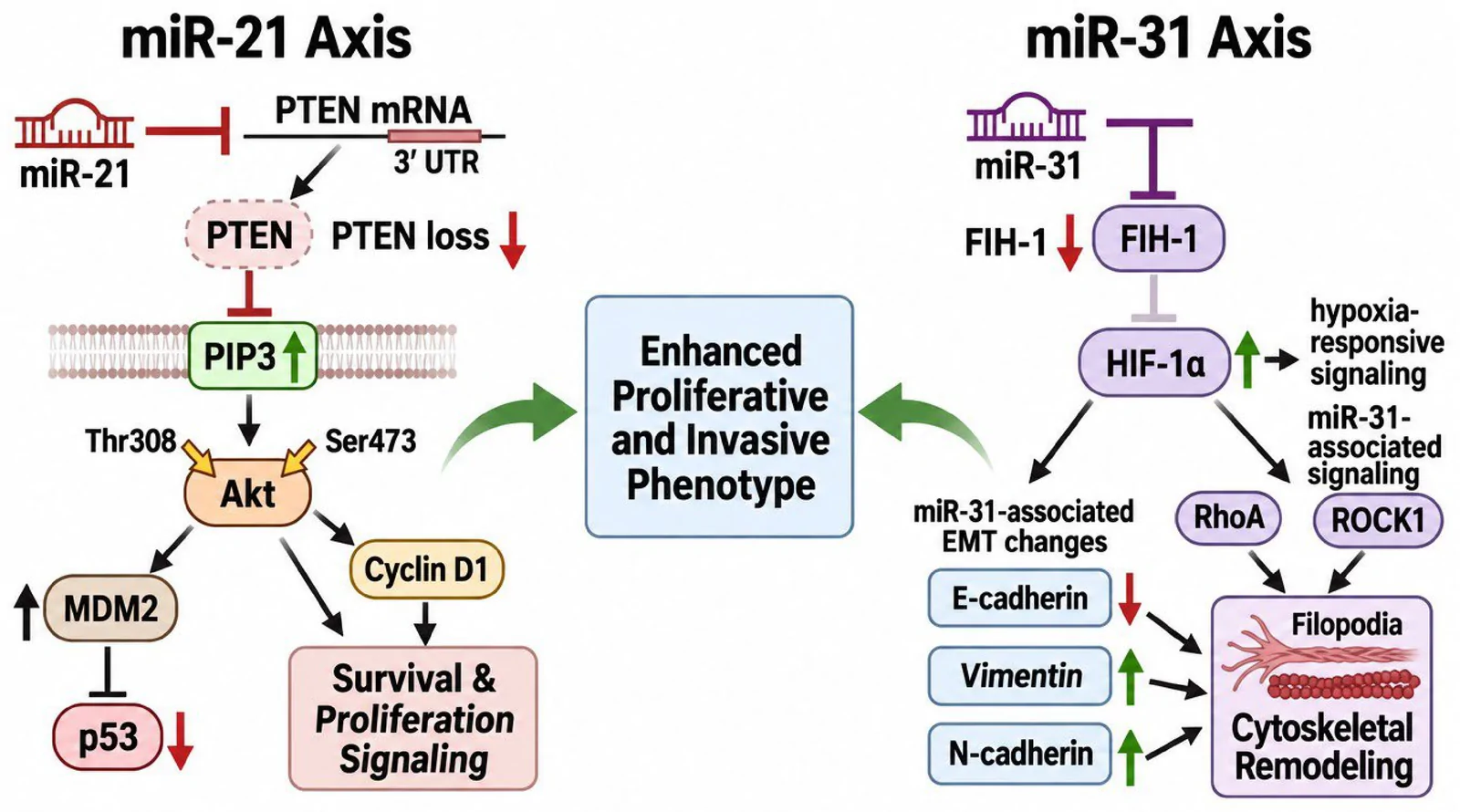
Mechanistic signaling cascades governed by miR-21 and miR-31 in Oral Squamous Cell Carcinoma. The diagram illustrates the reciprocal regulation of proliferative survival (via PTEN/Akt) and invasive remodeling (via FIH-1/RhoA), leading to an aggressive malignant phenotype.

Figure 1 summarizes the partly distinct but convergent molecular effects of miR-21 and miR-31 in OSCC, highlighting their respective associations with proliferative, survival, EMT-related, and invasive signaling pathways.

## 4. Salivary miR-21 and miR-31 as Diagnostic Biomarkers

Tumor-associated miRNAs in the salivary matrix originate from multiple biological processes. Although much attention focuses on the active secretion of microRNAs within extracellular vesicles (EVs), particularly exosomes (30–150 nm), it is important to recognize that not all salivary miR-21 and miR-31 are exosomal. Significant contributions also arise from the passive release of molecular cargo during tumor-associated apoptosis and necrosis [110,111,112]. Furthermore, saliva’s proximity to the oral tumor microenvironment allows direct shedding from the malignant epithelium [47,113,114]. A secondary, though less significant, contribution may also arise from the circulation via the salivary gland capillary network.

These markers retain their analytical utility due to their remarkable biophysical stability. Beyond exosomal sequestration, which provides a protective lipid bilayer, these miRNAs are often associated with argonaute-2 (AGO2) proteins or high-density lipoproteins [115,116,117]. This association shields the 22-nucleotide RNA fragments from the high concentrations of endogenous and exogenous RNases in the oral cavity. This dual-layer protection ensures the tumor’s molecular signature remains intact long enough for detection [118,119,120]. A systematic review of the literature indicates that salivary miR-21 is among the most reproducible indicators of OSCC [55,121,122,123]. Across multiple independent cohorts, miR-21 shows consistent and significant upregulation in the saliva of OSCC patients compared with healthy controls [44]. Across published qPCR-based studies, reported diagnostic performance for salivary miR-21 and miR-31 has generally been favorable, although sensitivity, specificity, and AUC vary considerably between cohorts and analytical protocols.

Systematic evaluation suggests that both markers are consistently upregulated in OSCC patients compared with healthy controls, but the magnitude of this change remains heterogeneous across the literature. Consequently, reported diagnostic accuracy must be interpreted within the context of varying salivary collection methods and the current lack of standardized endogenous controls for normalization. A critical requirement for any screening biomarker is the ability to distinguish OSCC not only from healthy tissue but also from clinically relevant controls, including OPMDs and inflammatory conditions. Evidence suggests that miR-21 and miR-31 are elevated in dysplastic OPMDs, but their expression is significantly higher in established OSCC, potentially distinguishing premalignant from established malignant disease [124,125,126]. However, generalized oral inflammation confounds the results, so any diagnostic panel must be calibrated to prioritize oncogenic specificity over generalized cellular stress. For a molecular tool to provide a clinical advantage over conventional oral examination, it must maintain analytical sensitivity in Stage I and II tumors. miR-21 and miR-31 dysregulation has been reported in early-stage disease, with several studies suggesting maintained sensitivity in early-stage subgroups [127,128]. However, the field currently lacks large-scale evidence from asymptomatic high-risk populations to confirm whether these markers can reliably identify silent malignant shifts before they become morphologically obvious. Evaluating miR-21 and miR-31 as a combined panel is biologically justified by their complementary roles: miR-21 reflects oncogenic survival, and miR-31 reflects epithelial dysregulation. While this biological complementarity is compelling, clinical synergy must be established through trials demonstrating a statistically significant improvement in AUC compared to single-marker assays. Any gain in diagnostic performance must also be large enough to justify the added analytical complexity of multiplex detection in a chairside setting.

## 5. Methodological Heterogeneity in Salivary miRNA Analysis

The physiological concentration of salivary miRNAs is strongly influenced by host-related variables and pre-analytical protocols, creating substantial biological noise that can obscure the oncogenic signal [129,130]. Chronic exposure to tobacco, alcohol, and areca nut, along with chronic periodontitis, induces generalized mucosal stress and immune activation that can independently upregulate miR-21 and miR-31 [131,132]. Consequently, not every surge in these markers indicates malignancy, so a diagnostic framework must account for local inflammatory status. Inconsistent collection and storage procedures further compound this biological variability. Pre-analytical choices, specifically the use of stimulated versus unstimulated saliva, directly affect exosomal concentration; mechanical or chemical stimulation increases salivary flow, which may dilute the detectable molecular cargo [133,134]. Furthermore, the salivary matrix is enzyme-rich, and delayed centrifugation or improper storage temperatures can facilitate rapid RNA degradation by endogenous RNases [135,136,137,138]. While exosome-encapsulated miRNAs are relatively stable, repeated freeze–thaw cycles can disrupt vesicular integrity, leading to cargo release and subsequent degradation, thereby hindering inter-study comparability across the literature [139].

A lack of standardization in the analytical and post-analytical phases of the diagnostic pipeline also stalls the clinical translation of salivary biomarkers [140]. RNA recovery efficiency depends heavily on extraction chemistry and salivary input volume. Variability among total RNA extraction protocols and those optimized for small-RNA fractions can reduce analytical sensitivity, particularly in early-stage OSCC, where target abundance is inherently low [141,142]. Furthermore, the lack of a universally accepted normalization strategy, often called the normalization paradox, remains a major barrier to reconciling fold-change data across cohorts. Historically used controls, such as RNU6B (U6) are increasingly scrutinized, as their suitability as stable extracellular references in saliva remains controversial [143]. While alternative strategies, such as synthetic exogenous spike-ins or stable endogenous miRNAs, provide some technical control, they do not necessarily correct for underlying biological variation in saliva production [144,145,146].

These methodological differences are directly evident across the clinical studies evaluating salivary miR-21 and miR-31. Zahran et al. used SNORD68 for normalization, whereas Scholtz et al. used SNORD60; Garg et al. normalized miR-21 expression to U6, while studies evaluating salivary miR-31 by Liu et al. and Syed et al. used miR-16 as an endogenous reference. Sample preparation was similarly heterogeneous: several studies collected unstimulated whole saliva, whereas others performed centrifugation and analyzed clarified salivary fractions or supernatant. RNA recovery methods also varied between commercial small-RNA isolation systems, phenol-based procedures, and column-based purification. Importantly, the core clinical studies evaluating salivary miR-21 and/or miR-31 predominantly relied on relative RT-qPCR measurements; purified extracellular-vesicle fractions and absolute quantification were not routinely employed. Thus, differences in reported miRNA abundance and diagnostic performance may reflect not only biological variation among cohorts but also differences in sample fraction, RNA recovery, and normalization strategy.

**Figure 2:**
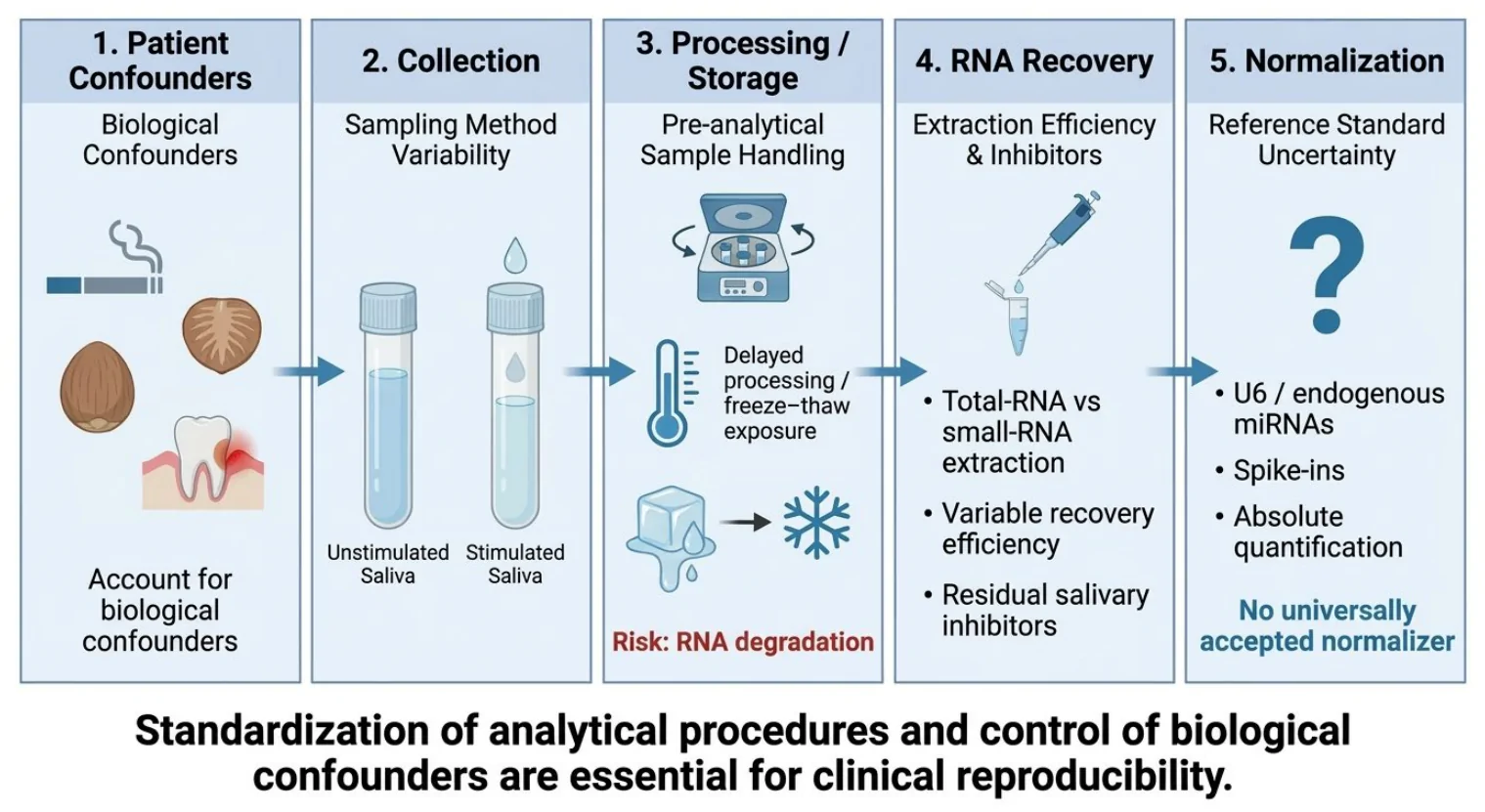
Systematic challenges in the salivary miRNA diagnostic pipeline. Critical variables including patient-related confounders, sampling inconsistencies, and the lack of standardized normalization protocols represent significant barriers to clinical reproducibility.

This technical heterogeneity also extends to diagnostic decision thresholds, where many studies derive thresholds from ROC curves optimized for small-scale, retrospective cohorts. This often leads to statistical overfitting, in which high AUC values fail to translate to external validation populations [147,148,149,150]. Until standardized procedures for collection, extraction, and normalization are established and diagnostic thresholds are externally validated, these molecular markers remain confined to research settings rather than clinical decision-making. The principal biological, pre-analytical, analytical, and normalization-related sources of variability across the salivary miRNA workflow are summarized in Figure 2.

## 6. From PCR-Based miRNA Detection to Isothermal RPA

### 6.1. Comparative Analysis of Current Molecular Benchmarks

The analytical landscape for miRNA detection is dominated by quantitative Polymerase Chain Reaction (qPCR), yet its clinical utility for rapid near-patient testing is inherently limited [151]. While RT-qPCR remains a widely used reference method because of its exceptional sensitivity and extensive quantitative literature, its reliance on precise thermal cycling and multistep sample preparation generally requires centralized laboratory infrastructure. Repeated denaturation and annealing cycles increase instrumentation requirements and complicate implementation within a rapid near-patient workflow. Furthermore, salivary components such as mucins and complex proteins can interfere with downstream enzymatic reactions, often necessitating RNA purification before amplification [152,153,154].

In contrast, digital PCR (dPCR) provides high-precision absolute quantification, particularly in heterogeneous biofluids. By partitioning the sample into many independent reaction compartments, dPCR reduces reliance on endogenous reference genes and improves quantitative resolution at low target abundance [155,156,157]. However, its instrumentation requirements and workflow complexity currently limit routine point-of-care deployment. Similarly, while Next-Generation Sequencing (NGS) is invaluable for biomarker discovery and transcriptomic profiling, its cost, analytical complexity, and turnaround time are incompatible with immediate near-patient molecular assessment [158,159,160]. Consequently, decentralized amplification designs must maintain sufficient analytical sensitivity and specificity while substantially reducing hardware requirements.

RPA is attractive in this context because it reduces dependence on thermal-cycling instrumentation. However, the technological feasibility of RPA should be distinguished from target-specific clinical validation. At present, RPA-based short-RNA detection has been demonstrated in proof-of-concept systems, but a clinically validated RPA assay for salivary miR-21 and/or miR-31 in OSCC has not been established.

### 6.2. The miRNA Length–Primer Architecture Conflict

A central technical obstacle to adapting mature miRNA detection to Recombinase Polymerase Amplification (RPA) is the mismatch between target length and primer architecture. Mature miRNAs are exceptionally short, typically ~19–24 nucleotides, whereas RPA primers are generally considerably longer, with approximately 30–35 nucleotides commonly recommended for efficient recombinase-mediated target recognition [161,162,163]. Because the target itself may be shorter than an individual RPA primer, conventional two-primer amplification cannot readily be implemented without first introducing additional sequence space for independent primer binding [164]. This challenge is compounded by the need to preserve sequence specificity under isothermal conditions. Unlike PCR, which separates denaturation and temperature-controlled primer annealing into discrete cycling steps, RPA recognizes targets continuously at a relatively low, constant temperature [165,166,167]. For highly homologous short RNA targets, this places substantial demands on primer and probe design, particularly when discrimination among mature miRNAs, closely related sequences, isomiRs, precursor species, or single-base variants is required [168,169,170]. Consequently, mature miRNAs generally need to be converted or extended into longer, amplifiable templates before RPA can be applied reliably [171,172,173].

### 6.3. Sequence-Extension Strategies: Bridging the Gap

To overcome the limited sequence space of mature miRNAs, several target-extension strategies can generate longer, amplifiable templates. Stem-loop Reverse Transcription (RT) uses a structured primer that recognizes the 3′ end of the mature miRNA and adds extra sequence during cDNA synthesis. This approach can improve discrimination between mature and precursor species but requires target-specific primer design and an additional enzymatic step [174,175,176]. Alternatively, Poly(A) Tailing adds a polyadenosine sequence to the 3′ end of the miRNA, followed by reverse transcription using an oligo-dT-containing primer [177,178]. This strategy provides a more universal route to template extension but requires sufficient downstream primer or probe specificity to distinguish the intended target from other polyadenylated or extended RNA species. Additional approaches include Adapter Ligation, in which defined oligonucleotide sequences are ligated to one or both termini of the miRNA, and Target-Specific RT Extension, in which additional sequence information is introduced during reverse transcription [179,180,181]. Experimental studies have shown that mature miRNAs can be converted into RPA-compatible templates. For example, RT-RPA-based systems have demonstrated detection of converted miRNA targets such as miR-155, supporting the general feasibility of adapting short mature miRNAs to RPA architectures. More recent proof-of-concept work has also reported RPA-compatible detection of miR-21 following target conversion. However, these studies were not performed as salivary OSCC assays, and equivalent experimental validation for salivary miR-31 was not identified. Accordingly, the application of these conversion strategies to salivary miR-21/miR-31 in OSCC remains prospective rather than clinically established.

For near-patient implementation, the optimal architecture must therefore balance target specificity, conversion efficiency, reaction time, and compatibility with a highly integrated isothermal workflow. The translational challenge is therefore not only to generate an RPA-compatible template, but also to integrate target conversion and amplification in a manner that minimizes enzyme incompatibility, manual handling, and total assay time.

### 6.4. RPA Mechanism: Enzymatic Co-Operation at Constant Temperature

Recombinase Polymerase Amplification replaces repeated thermal cycling with enzyme-mediated target recognition and strand displacement [182,183]. Recombinase proteins associate with primers to form nucleoprotein complexes that search double-stranded DNA or cDNA for homologous sequences. After target recognition, the primer invades the duplex, while accessory proteins stabilize the displaced strand and prevent rapid re-annealing. A strand-displacing DNA polymerase then extends the bound primer, generating new DNA without the high-temperature denaturation step required in conventional PCR [162,184,185,186,187].

Because these reactions proceed efficiently at relatively low, constant temperatures, typically around 37–42 °C, RPA substantially reduces hardware requirements compared with conventional thermal cycling [188]. This characteristic, together with rapid amplification kinetics and compatibility with dried reagent formats, makes RPA attractive for decentralized molecular testing, provided that assay specificity, contamination control, and compatibility with upstream miRNA-conversion steps are adequately addressed [189,190,191,192,193].

Importantly, rapid amplification kinetics should not be equated with the turnaround time of a complete clinical assay. A sample-to-answer workflow would also need to include saliva preparation, RNA release or extraction, miRNA conversion, amplification, signal detection, and result interpretation.

### 6.5. Analytical Limitations and Clinical Reliability

Despite its advantages, RPA presents several technical limitations that must be addressed for reliable miRNA detection. A major challenge is maintaining sequence specificity, particularly for short and highly homologous RNA targets [194]. Primer–primer interactions, off-target amplification, and tolerance of sequence mismatches can compromise analytical performance when assay design is suboptimal [195]. Carryover contamination is another important concern. RPA rapidly generates large quantities of amplicon, and transfer of previously amplified material into subsequent reactions can produce false-positive signals [196,197,198]. Closed reaction systems that minimize post-amplification handling are therefore particularly important for clinical implementation [199].

Quantitative interpretation also remains more challenging than in established qPCR or dPCR workflows because RPA amplification kinetics can become nonlinear and rapidly approach saturation [200,201]. Multiplex detection introduces additional complexity, as multiple primer sets may compete within the same reaction and alter amplification efficiency [202]. If miR-21 and miR-31 were ultimately evaluated within the same assay, physically separated reaction zones or other compartmentalized architectures may therefore represent a practical alternative to single-volume multiplex amplification.

Ultimately, RPA addresses an important hardware limitation of conventional molecular diagnostics. Still, its clinical value for salivary miRNA detection will depend on demonstrating sufficient sensitivity, sequence specificity, reproducibility, resistance to matrix-associated inhibition, and effective contamination control within an integrated sample-to-answer workflow.

## 7. Translational Requirements for a Near-Patient RPA Workflow

The translational value of salivary miR-21 and miR-31 ultimately depends on whether their detection can be integrated into a rapid, reproducible, and clinically interpretable workflow outside a centralized molecular laboratory. Such translation requires more than replacing thermal cycling with isothermal amplification; it requires coordinated optimization of sample preparation, miRNA conversion, amplification, contamination control, readout, and clinical interpretation within a simplified sample-to-answer platform.

### 7.1. Rapid Saliva Preparation and the Lysis-to-Amplification Barrier

Sample preparation remains a major bottleneck in developing a near-patient molecular assay. Conventional salivary miRNA workflows rely on centrifugation, RNA purification, and multiple handling steps that are poorly suited to point-of-care use [203,204]. A clinically practical system would therefore require simplified RNA-release or extraction strategies that recover free, protein-associated, and vesicle-associated miRNAs while remaining compatible with downstream enzymatic reactions. The principal challenge is balancing efficient miRNA release with preservation of RPA activity. Salivary proteins, mucins, nucleases, and other matrix components may interfere with reverse transcription or amplification [205,206]. Consequently, rapid lysis and extraction-minimized approaches must be evaluated not only for RNA recovery but also for inhibitor tolerance, reproducibility, and compatibility with subsequent target-conversion and amplification steps.

### 7.2. miRNA Conversion and RPA Assay Architecture

**Figure 3:**
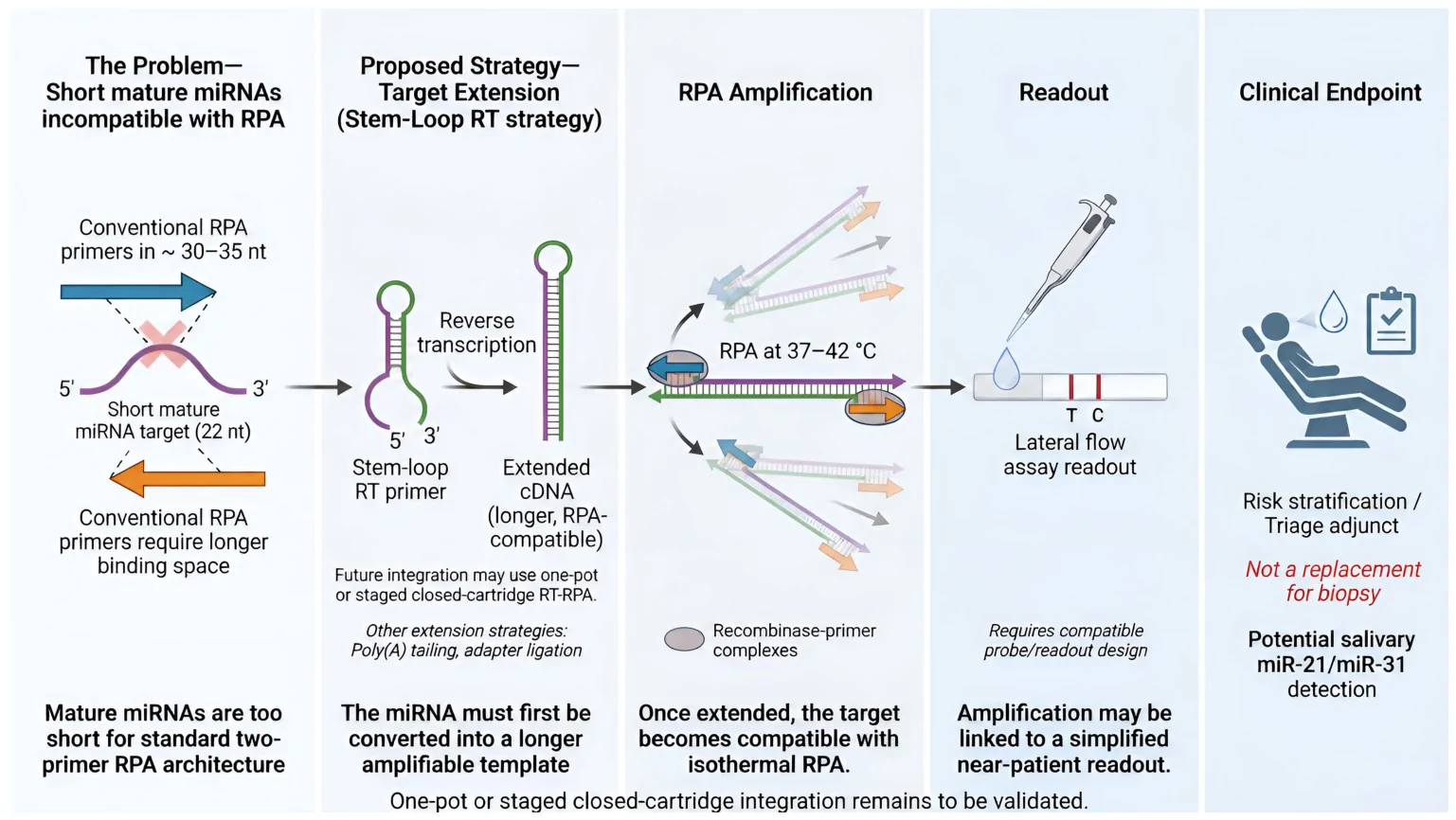
Conceptual adaptation of mature miRNA targets to an RPA-compatible near-patient workflow. Mature miRNAs require prior target conversion or sequence extension to generate longer cDNA templates suitable for RPA. Following isothermal amplification, products may potentially be coupled to simplified readout formats such as fluorescence or lateral-flow detection. The workflow shown is conceptual and has not yet been clinically validated for salivary miR-21/miR-31 detection in OSCC. Its proposed clinical role is adjunctive risk stratification and triage of patients with clinically suspicious oral lesions rather than population screening or replacement of histopathological diagnosis.

Because mature miRNAs are too short for conventional RPA primer architecture, an upstream conversion or extension step is required [207,208]. Candidate approaches include stem-loop reverse transcription, poly(A)-mediated extension, adapter ligation, and target-specific reverse transcription strategies. For future near-patient use, highly integrated RT/extension-RPA architectures are particularly attractive because they could reduce manual handling and the risk of contamination. However, integration must not compromise discrimination between mature miRNAs and precursor or closely related RNA species [209,210,211,212]. The optimal architecture must therefore balance specificity, conversion efficiency, reaction time, enzyme compatibility, and operational simplicity. For point-of-care implementation, a one-pot RT-RPA format would be attractive because it could reduce manual transfer and contamination risk. However, simultaneous target conversion and RPA may be limited by differences in optimal temperature, buffer composition, divalent-ion requirements, and reaction kinetics between reverse-transcription/extension enzymes and the RPA enzyme system. Such enzyme crosstalk could reduce conversion efficiency or amplification performance. A practical alternative may therefore be a temporally staged single-cartridge design, in which target extension is completed first and RPA proceeds subsequently within the same closed system without manual transfer. Importantly, total turnaround time must include saliva preparation, target conversion, amplification, and readout rather than the amplification step alone. Although a sub-30-min workflow would be desirable for chairside use, this should presently be regarded as a development objective rather than an experimentally demonstrated performance for salivary miR-21/miR-31 detection. Although converted-miRNA RPA has been demonstrated experimentally for selected targets, a complete conversion-to-RPA workflow has not yet been clinically validated for salivary miR-21/miR-31 detection in OSCC. Figure 3 therefore represents a conceptual translational pathway rather than an established assay.

### 7.3. Multiplex Detection and Internal Controls

If future studies demonstrate that combined measurement of miR-21 and miR-31 provides added diagnostic value, simultaneous amplification within a single reaction may introduce primer competition and differential amplification efficiencies [213]. Physically separated reaction zones within a single cartridge may therefore offer a pragmatic first-generation approach while preserving a unified clinical workflow. Equally important is the inclusion of internal controls. Process controls should confirm successful sample preparation and target conversion, while amplification controls should identify reaction failure or salivary inhibition. Without such controls, a negative signal cannot be confidently interpreted as true absence of the biomarker.

### 7.4. Readout and Closed-System Integration

RPA is compatible with several simplified detection formats, including fluorescence-based and lateral-flow readouts [214,215,216]. For clinical implementation, the preferred format should minimize post-amplification handling because opening reaction vessels after amplification substantially increases the risk of carryover contamination. Closed-cartridge architectures that integrate sample preparation, target conversion, amplification, and signal detection may therefore be particularly suitable for future near-patient testing. The optimal readout should provide unambiguous interpretation while maintaining sufficient sensitivity and specificity for clinically relevant target concentrations [217].

However, no complete sample-to-answer salivary miR-21/miR-31 RPA workflow with a demonstrated 30-min turnaround time has been established. Accordingly, turnaround-time claims should not be based on the RPA amplification step alone and should only be made after experimental evaluation of the complete workflow.

### 7.5. Clinical Positioning and Validation Requirements

The intended clinical-use scenario considered in this review is adjunctive risk stratification and triage of patients who already present with a clinically suspicious oral lesion. In this setting, a salivary miRNA assay would not establish a definitive diagnosis or replace histopathological biopsy. Rather, it could potentially provide additional objective molecular information to support referral, prioritization, and selection of patients requiring definitive diagnostic evaluation. This proposed use should be distinguished from population screening of asymptomatic individuals, surveillance of high-risk individuals without a clinically apparent lesion, or prediction of malignant transformation in patients with OPMDs. These applications represent distinct clinical questions and would require dedicated prospective validation in their respective target populations. Current evidence for salivary miR-21 and miR-31 is insufficient to support such broader screening claims. Before clinical implementation, analytical validation must establish the limit of detection, sequence specificity, reproducibility, matrix tolerance, and contamination control. Clinical validation should then assess performance in independent, preferably multicenter cohorts that include early-stage OSCC, oral potentially malignant disorders, inflammatory oral disease, benign lesions that clinically resemble OSCC, and other relevant comparator groups. Only after such analytical and clinical validation can the practical value of an RPA-based salivary miRNA triage assay be determined.

## 8. Conclusions

Salivary miR-21 and miR-31 are biologically relevant to OSCC and have been repeatedly reported as OSCC-associated salivary biomarkers in clinical studies. Their involvement in pathways associated with malignant progression, together with their detectability and relative stability in saliva, provides a rationale for further evaluation as adjunctive biomarkers. However, differences in saliva collection, RNA recovery, normalization, comparator groups, and diagnostic thresholds continue to limit reproducibility and direct comparison across studies. The added diagnostic value of combining miR-21 and miR-31 remains uncertain. Although their partly distinct biological roles provide a rationale for joint evaluation, current evidence does not establish that a combined miR-21/miR-31 panel performs better than either marker alone. Demonstration of diagnostic synergy will require direct within-cohort comparison followed by independent validation in clinically relevant populations. Isothermal amplification technologies such as Recombinase Polymerase Amplification (RPA) remain a prospective translational approach rather than an established clinical method for these targets. Although RPA-based systems can support detection of converted miRNA templates, a clinically validated sample-to-answer assay for salivary miR-21 and/or miR-31 in OSCC has not yet been demonstrated. Future development will therefore require reliable target-extension strategies, adequate sequence specificity, resistance to salivary matrix interference, contamination control, reproducibility, and validation in patients with clinically suspicious oral lesions. Only after these analytical and clinical requirements are met could such an assay be considered for adjunctive near-patient triage and risk stratification rather than definitive diagnosis.

## Figures and Tables

**Table 1:** Clinical Evidence for Salivary miR-21 and miR-31 in OSCC.

Study	Population/Comparator	OSCC Stage	Saliva/Collection	RNA Extraction	Detection/Normalization	Marker Result	Diagnostic Threshold/Performance	Independent Validation
Zahran et al., 2015 [39]	20 OSCC; 40 OPMD (20 dysplastic, 20 non-dysplastic); 20 recurrent aphthous stomatitis; 20 healthy controls	NR	Unstimulated saliva; participants refrained from eating, drinking, or chewing gum before collection; saliva supernatant	microRNA Isolation Kit (Qiagen)	qRT-PCR SNORD68	miR-21 ↑ in OSCC and OPMD vs healthy/disease controls	OPMD vs healthy/disease controlsmiR-21 cutoff: ~4-fold increase; Sensitivity 65%; Specificity 65%	No independent validation reported
Yap et al., 2018 [40]	30 OSCC oral swirls; 30 controls; training 15/15 and separate test 15/15	Mixed/NR in diagnostic analysis	Oral-swirl/salivary sample; centrifuged before RNA extraction	mirVana microRNA isolation Kit	RT-qPCR	miR-21 detectable and upregulated; salivary miR-31 was not consistently detectable in oral swirls	MultimiRNA dysregulation score: AUC 0.95 training; AUC 0.86 test. Test classification identified 15/15 OSCC and 10/15 controls correctly. Values are for the panel, not miR-21 alone	Yes—internal test cohort, but no external cohort
Kumari/Syed et al., 2021 [41]	19 OSCC patients sampled before and after surgery; 2 healthy calibrator controls; 16 paired patients remained after RNA-quality exclusions	Various stages; clinicopathological correlations assessed	Fasting, unstimulated whole saliva, PureSAL device; stored at −80 °C	mirVANA PARIS kit	TaqMan RT-qPCR; miR-16 endogenous control	miR-31 expression assessed pre- vs postoperative OSCC	No validated OSCC-vs-control diagnostic cutoff, sensitivity, specificity, or AUC reported	No
Scholtz et al., 2022 [42]	43 OSCC; 44 controls	Mixed stages	Whole unstimulated saliva; multicenter collection; standardized pre-collection conditions	Total RNA isolated after standardized saliva processing	TaqMan RT-qPCR; SNORD60	miR-31-5p significantly ↑ in OSCC; miR-21-5p not significantly different between OSCC and controls	Individual miRNAs showed limited discrimination; three-marker miR-31/miR-345/miR-424 model: AUC 0.87, sensitivity 0.86, specificity 0.77. Do not attribute these values to miR-31 alone.	No independent external validation reported
Jadhav et al., 2022 [43]	130 OSCC patients; comparison based on presence/absence of cervical lymph-node metastasis	Multiple TNM stages	Unstimulated whole saliva	NR in accessible abstract	Real-time PCR	Higher salivary miR-21-5p/3p associated with nodal metastasis	For salivary miR-21-5p: cutoff 2.32 Ct/fold-change measure, Sensitivity 42.6%, Specificity 90.3%; miR-21-3p: cutoff 2.16, Sensitivity 60.3%, Specificity 83.9%. This is nodal-metastasis prediction, not OSCC-vs-control diagnosis.	No independent external validation reported
Garg et al., 2023 [44]	30 OSCC; 30 OPMD; 30 healthy controls	OSCC: Stage II 1; III 3; IVa 24; IVc 2; no Stage I	whole unstimulated saliva	RNA isolated from saliva	qRT-PCR; U6 reference	miR-21 ↑: OSCC > OPMD > control	OSCC vs control: cutoff 6.79-fold, Sensitivity 80%, Specificity 70%; OSCC vs OPMD: cutoff 2.2-fold, Sensitivity 70%, Specificity 60%	No independent validation reported

(NR = Not Reported).

## Data Availability

No new data were created or analyzed in this study. Data sharing is not applicable to this article.
